# Individualized pleasure-oriented exercise sessions, exercise frequency, and affective outcomes: a pragmatic randomized controlled trial

**DOI:** 10.1186/s12966-024-01636-0

**Published:** 2024-08-05

**Authors:** Diogo S. Teixeira, Vasco Bastos, Ana J. Andrade, António L. Palmeira, Panteleimon Ekkekakis

**Affiliations:** 1 Faculty of Physical Education and Sport, Lusófona University, Lisbon, Portugal; 2 Research Center in Sport, Physical Education, and Exercise and Health (CIDEFES), Lisbon, Portugal; 3 Michigan State University, Michigan, USA

**Keywords:** Exercise, Adherence, Pleasure, Affect, Motivation

## Abstract

**Background:**

Affective responses are increasingly recognized as potentially effective intervention targets that may facilitate exercise and physical activity behavior change. While emerging correlational evidence suggests that more pleasant affective responses are associated with higher participation and adherence, experimental evidence remains scarce. In light of this, we conducted a preregistered, pragmatic, single-blinded, superiority randomized controlled trial with two parallel groups, with the goal of determining the impact of an individualized exercise-intensity prescription targeting pleasure on exercise frequency.

**Methods:**

Forty-seven non-regular exercisers were randomized into two groups. For both groups, the intervention consisted of three exercise sessions based on the Frequency-Intensity-Time-Type (FITT) principle. However, the experimental group also received an individualized intensity prescription based on prior assessment of preference for and tolerance of exercise intensity, as well as instructions emphasizing the promotion of pleasure as a basis for self-regulating exercise intensity. The primary outcome was gymnasium attendance over an eight-week follow-up period. Secondary outcomes were affective valence and arousal, post-exercise enjoyment, core affective exercise experiences, and anticipated and remembered affect.

**Results:**

Forty-six participants were retained for analysis (M_age_ = 32.00; SD = 8.62 years; 56.5% female). Compared to the control group, the experimental group exhibited 77% higher session attendance (14.35 vs. 8.13 sessions) over the eight-week follow-up period (group main effect *p* = 0.018, η^2^_p_ = 0.120; Cohen’s *d* ranged from 0.28 to 0.91 during follow-up). Also, the experimental group reported higher levels of pleasure during the intervention sessions (for all group main effects, *p* < 0.001, η^2^_p_ from 0.33 to 0.37) and higher levels of remembered pleasure (group main effect *p* = 0.021, η^2^_p_ = 0.116) and anticipated pleasure (group main effect *p* = 0.022, η^2^_p_ = 0.114). No harm was detected.

**Conclusions:**

These results demonstrate the practicality and effectiveness of an intervention aimed at enhancing affective responses to exercise in improving short-term session attendance.

**Trial registration:**

ClinicalTrial.gov NCT05416593.

**Supplementary Information:**

The online version contains supplementary material available at 10.1186/s12966-024-01636-0.

## Background

Researchers from the World Health Organization have estimated that, if the prevalence of physical inactivity does not change by 2030, there will be approximately 500 million new cases of preventable major non-communicable diseases globally [70]. The category of major non-communicable diseases includes coronary heart disease, stroke, type 2 diabetes, several types of cancers, depression, and dementia. The economic burden of failing to increase physical activity by 2030 is estimated at over half a trillion dollars. Taken together, these estimates underscore the need to increase population-level physical activity rates. However, as the editors of *The *Lancet [50] noted, “since 2001, there has been no improvement in global levels of physical activity” (p. 365). Experts are increasingly characterizing efforts to promote physical activity as a failure (e.g., [16, 36, 40, 61]).

The cornerstone of interventions to promote physical activity is the idea that, as rational beings, humans are inherently interested in enhancing or preserving their health. Therefore, it is generally presumed that providing people with compelling information that physical activity will benefit their health should constitute a powerful motivational impetus for them to initiate and maintain a physically active lifestyle. However, the emphasis on health promotion as the central motivational argument has not borne fruit (e.g., [79]). For example, among individuals with known risk factors for cardiovascular disease, a meta-analysis [55] of 30 randomized controlled trials (*N* = 19,834) examining the effect of behavioral counseling interventions administered within healthcare settings (i.e., focusing on health promotion as the central argument) found a pooled standardized mean difference (SMD) in physical activity of 0.06 (95% confidence interval from -0.03 to 0.14). In healthy adults, another meta-analysis [60] of 59 randomized controlled trials (*N* = 20,801), also evaluating behavioral counseling interventions in the context of primary care yielded a SMD of 0.19 (95% confidence interval from 0.14 to 0.25). To explain the meaning of SMD = 0.19, this number is equivalent to a 92.4% overlap between the distributions of the treatment and control groups. Another way to interpret the meaningfulness of SMD = 0.19 is by calculating the “probability of superiority,” namely the probability that a person picked at random from the intervention group would exhibit a higher level of physical activity than a person picked at random from the control group [67, 68]. SMD = 0.19 entails that this probability barely exceeds chance (i.e., 55.3%. A third way to put a SMD = 0.19 into perspective is to point out that the magnitude of the so-called “question-behavior effect” (i.e., the apparent increase in “physical activity” one can expect by simply asking respondents to complete measures of physical activity at two time points is estimated as SMD = 0.21 (95% CI from 0.08 to 0.34, see [54]). It is also important to note that, although meta-analyses show that counseling interventions in healthcare contexts may increase moderate-to-vigorous physical activity by 9.1 min per week [55] or 33.0 min per week [60], these figures reflect assessments of physical activity based on self-reports. On the other hand, when physical activity is assessed by mechanical devices (i.e., accelerometers, pedometers), the changes in physical activity are attenuated and become trivial and not significantly different from zero (e.g., 4.1 min per week, see [48]).

Beyond cultivating outcome expectations (i.e., beliefs that participation in regular physical activity will benefit health), interventions based on psychological theories generally assume that physical activity is the natural consequence of a deliberative behavioral decision-making process. In turn, deliberative decisions are assumed to rely on cognitive appraisals. These appraisals may include beliefs about the likelihood that engagement in the behavior will, in fact, yield the desired (health, fitness, weight-loss, or other) benefits, the value that the individual places on these benefits, beliefs about the possible ramifications of inaction, beliefs about personal capabilities to enact the behavior, beliefs about the likelihood of identifying sources of social support, beliefs about the likelihood of receiving praise or encouragement from important others, and beliefs about whether the social environment will support the basic needs to experience autonomy, a sense of competence, and the formation of meaningful social bonds. Although these factors seem to be plausible mediators of behavior change, and therefore reasonable targets of interventions, the evidence to date suggests that the ability of interventions based on psychological theories to influence behavior is minimal and any noticeable changes in behavior are only weakly mediated by these psychological constructs [63].

Thus, there is a growing sentiment among experts that there is a “need for new thinking” [40], p. 650) because “more of the same is not enough” [35], p. 190). More specifically, it has been argued that “to advance the field, we need research to identify novel targets for physical activity behavior change interventions that leverage basic behavioral science principles beyond cognitive processes” [18], pp. 140–141). Arguably, the most notable trend that has emerged over the last several years in response to calls for new ideas has been the rediscovery of the time-honored idea that affective factors, such as various forms of pleasure and displeasure, can be powerful motivators of human behavior [20]. This trend within the exercise sciences has coincided with what has been called “the rise of affectivism” in the behavioral sciences [17].

The interest in affective experiences as possible motivators of exercise and physical activity behavior has sparked a recent wave of exercise-specific motivational theories [7, 14, 73, 74]. The theories that have been proposed thus far fall under the general rubric of “dual-process theories.” Although they vary in some of their details, these theories are based on the fundamental premise that behavior is not solely under the control of cognitive processes but, instead, represents the result of a dynamic interaction between cognitive/deliberative and automatic/affective processes, with the important recognition that the two classes of processes “will not always converge and, in some cases, may be discordant” [14], p. 231). For example, individuals may recognize exercise as a health-promoting behavior, may perceive ample support from their social environment, may feel confident in their ability to physically carry out the behavior, but may remain hypoactive due to a lifetime of unpleasant affective experiences, ranging from feeling embarrassed by critical comments during physical education to viewing public gymnasia as intimidating.

For example, according to the Affective-Reflective Theory (ART) of exercise and physical inactivity [7, 21], repeated episodes of pleasant or unpleasant experiences from exercise, over time, will consolidate a positive or negative “affective valuation” of the stimulus-concept of “exercise.” Future encounters with this concept will automatically trigger the attendant affective state (e.g., displeasure) and associated action tendency (e.g., avoidance). Moreover, because these affective processes are extremely efficient (i.e., do not require slow executive processing and thus unfold quickly and effortlessly), they precede and, therefore, influence the cognitive (or “reflective”) processes that follow. Thus, the ART postulates that, for exercise to be consistently selected among alternative behavioral options, both the reflective and the affective processes must be congruent (i.e., both should be positive).

If this theoretical postulate is true, it suggests an important, but still relatively untapped, research agenda for exercise psychology, namely the evaluation of methods to make the experience of exercise more pleasant for as many segments of the population as possible. Ekkekakis and Brand [21] wrote that developing an evidence-supported repertoire of interventions “is a prerequisite before pragmatic randomized controlled trials can be conducted to examine whether improved affective experiences can ultimately lead to increased and sustained exercise and physical activity participation” (p. 39).

Modifying affective responses to exercise can be accomplished by targeting three categories of possible mediators. First, one can aim to change the exercise stimulus itself, including the physical and social environment in which exercise is embedded. For example, research has been conducted on the relation of affective responses to the exercise “dose” (primarily, its intensity), the ambient temperature and humidity, urban or “green” scenery, the presence of (potentially critical) observers, the presence of mirrors, and the behavior of exercise professionals and fellow exercisers [23]. Second, one can modify the perception of exercise-induced somatosensory stimuli. Studies have tested the idea of creating “sensory competition” through engaging audiovisual stimulation (e.g., [78]) or immersive virtual reality (e.g., [6, 44]) as a way of alleviating the impact of somatosensory cues associated with strenuous exercise (e.g., respiratory sensations, aching muscles, hyperthermia). Third, one can change the cognitive appraisal of exercise and its meaning for the individual. Relevant research has examined the role of appraisals of self-efficacy, competence, autonomy, outcome expectations, or reappraisals (i.e., reinterpretations) of the meaning of unpleasant somatosensory cues (e.g., [34]).

A recent systematic review found that several techniques “offer implementable strategies to change how people feel during exercise” [45], p. 24). However, some of these techniques rely on extrinsic technical means (e.g., music, video, virtual reality) and others may require a period of guided practice before they can be used effectively (e.g., reappraisal). On the other hand, a method that has long been identified as effective and does not require any technical means or prior practice is the instruction to participants to self-regulate their intensity [19, 81] or, more explicitly, to regulate their intensity in a manner that enables them to maintain a pleasant affective state [66].

Both the instruction to self-regulate exercise intensity (e.g., [33, 52, 59]) and the instruction to regulate exercise intensity to maintain a pleasant affective state (e.g., [15, 37, 56, 57, 66]) have been shown to accomplish the dual goal of (a) keeping exercise intensity within the “moderate-to-vigorous” range that is recommended for the enhancement and maintenance of cardiorespiratory fitness and health, while also (b) yielding higher ratings of pleasure compared to traditional methods of exercise-intensity prescription (e.g., instructions to exercise within a target heart-rate zone). In fact, it has been observed that allowing participants to self-regulate their intensity can “uncouple” affective responses from the typical dose–response relationship linking (imposed) exercise intensity to affective responses, thereby making it possible to attain higher levels of intensity without incurring declines in pleasure [24]. Studies have also shown that self-regulation [83, 85] and affect-guided regulation of intensity [4] may result in individuals being willing to do more physical activity, while also producing fitness- and health-enhancing physiological adaptations [56, 57].

Similar results have been reported for resistance exercise. When individuals are instructed to self-select their training loads (e.g., [27, 31]) or are instructed to select loads that make them feel “good,” they choose loads within the range that is recommended to stimulate muscle hypertrophy and gains in strength [28]. Indeed, training programs involving self-selected loads have been effective in improving strength [29], especially among novice exercisers [72].

To facilitate the transition from research to practice, the present study was designed as a pragmatic, single-blinded, parallel-group, superiority randomized controlled trial (RCT) based on a previously published protocol [77]. Our intervention was designed as a brief (three-session), individualized exercise-intensity prescription aimed to enhance affective responses to exercise. The primary outcome of interest was gymnasium attendance over an eight-week follow-up period. We hypothesized that the experimental group would exhibit higher attendance during the follow-up period compared to the control group receiving a standard exercise prescription. In terms of secondary outcomes, we hypothesized that participants in the experimental group would report (a) more positive affective valence during the exercise sessions, and higher scores on (b) higher post-exercise enjoyment, (c) more pleasant core affective exercise experiences [26], (d) more pleasant anticipated affective response, and (e) more pleasant remembered affect.

## Methods

### Trial design

We conducted a pragmatic randomized, single-blinded, controlled superiority trial with two parallel groups and an allocation ratio of 1:1. The trial was preregistered (June 2022; NCT05416593) and was completed between August and November 2022. In addition, a comprehensive trial protocol has been published [77]. No notable changes occurred between the published trial protocol and the methods that were eventually implemented. The present report follows the CONSORT guidelines for transparent reporting [71] and later extension [12], and the respective checklist can be found in Supplementary file 1.

### Participants

Participants were deemed eligible if they were between 18 and 45 years old, were apparently healthy and without contraindications to exercise, and had a body mass index (BMI) between 18.5 and 30 kg/m^2^. The lower bound of the age range was dictated by the age of consent and the upper bound by risk-management considerations (i.e., age ≥ 45 years is considered a risk factor for men, according to the American College of Sports Medicine). Participants were also required to have previous experience in a health club setting but with a frequency of fewer than five workouts per month in the last six months (and no more than three months without attendance). Likewise, eligible participants could not have regular participation in organized sports five or more times per month in the last six months. Individuals were disqualified if they had a high-risk stratification for cardiovascular disease as specified by the American College of Sports Medicine [1] and/or exhibited high blood pressure (≥ 130/80 mmHg), as measured prior to the intervention. Additional exclusion criteria included an injury and/or inability to perform the exercise sessions without modifications and at the required time schedule (i.e., 48 h–96 h apart over two consecutive weeks). The study was conducted in two health clubs in the Lisbon district of Portugal. One site was a medium-sized health club in the suburbs of Lisbon and the other was a medium-sized club near the city center, geared primarily toward a more affluent clientele.

### Interventions

#### Pre-exercise evaluation and exercise program

All participants performed a pre-intervention session, to collect information on sociodemographic and exercise-psychological variables. An evaluation that included assessments of body weight, blood pressure, resting heart rate, and cardiovascular risk (PAR-Q + ; [8]) was also conducted. All participants subsequently performed three individualized sessions, consisting of the same exercise program, grounded on the Frequency-Intensity-Time-Type (FITT) principle, as specified by the guidelines of the American College of Sports Medicine [1]. However, the experimental group (FITT + AFFECT) received instructions aiming to promote an improved affective experience. To achieve this goal, the experimental intervention included the following components: (i) assessment of individual differences related to preference for higher or lower exercise intensity and higher or lower tolerance for high exercise intensity, and use of these data to advise exercisers regarding the initial choice of intensity, as well as subsequent upward adjustments in intensity; (ii) encouraging the self-regulation of intensity to ensure and maintain a positive affective experience; and (iii) repeated assessments of affective responses and use of these self-ratings as a basis for self-regulating exercise intensity. The control group (FITT) received standard instructions, in accordance with American College of Sports Medicine [1] guidelines. Both the experimental (FITT + AFFECT) and the control (FITT) interventions were delivered as ‘personal training,’ individualized sessions involving one trainer and one participant and appeared identical to outside observers. To further reduce the possibility of cross-treatment contamination, (a) verbal interactions were always in close physical proximity and (b) participants were unaware of the specific nature of the interventions being compared in the trial (i.e., whether or not the instructions they were receiving from their trainer were designed to enhance their affective experience).

The exercise sessions were structured in three parts, with a total duration of approximately 60 min: the initial phase consisted of 15 min of progressive aerobic activity on a treadmill (10 min at moderate + 5 min at vigorous intensity); the main phase comprised five resistance exercises (chest press, leg press, lat pulldown, leg curl, shoulder press), with 2 sets of 8–12 repetitions each, with 1 min rest between sets, and 2:0:2 cadence, followed by 15 min of aerobic activity of decreasing intensity on a stationary cycle (5 min at vigorous + 10 min at moderate intensity); the final phase consisted of four static passive stretching exercises (quadriceps, hamstrings, gluteus, triceps), performed in 2 sets of 20 s each. These sessions were conducted within a period of two weeks, with sessions spaced 48 h to 96 h apart. More details can be found in the study protocol [77].

#### Exercise session assessment timepoints

The variables of interest were measured prior to, during, and after each exercise session in both groups. Pre-exercise measurements (5–10 min before each session) included the anticipated affective response and remembered affect (referring to the previous session). During the session, heart rate was measured continuously. Also, the Feeling Scale (FS) and Felt Arousal Scale (FAS) were administered a total of 15 times at prespecified time points [3, 5, 41] as follows: at 5-min intervals during the initial aerobic activity (total of three times), immediately after the last set of each resistance exercise (total of five times), and at 5-min intervals during the aerobic activity during the main phase (total of three times); and in the last 10 s of the second set of each stretch in the final stage (total of four times). Post-exercise measurements took place at two points. The first occurred 5 min after the end of exercise in all sessions, assessing the enjoyment of the session; the second took place 24 h after each session to assess sleep, stress, fatigue, and muscle soreness. Additionally, for the last session, the anticipated affective response and remembered affect were assessed 48 h-96 h after each session (mimicking the previous inter-session intervals; the measurement of the anticipated affective response was in anticipation of a hypothetical fourth exercise session). All variables that were assessed at baseline were collected again after the last session (~ 10 min after the completion of the session). A diagram of the timing of assessments can be found in the study protocol [77].

### Outcomes

#### Primary outcome

Exercise session attendance during the eight weeks post-intervention was designated as the primary outcome of interest and was compared between groups and across time. Attendance data were obtained through the electronic record of the turnstile at the entrance of the gymnasia and cross-checked through regular individual questioning.

#### Secondary outcomes

Affective responses were assessed using the Portuguese versions [11] of the Feeling Scale (FS [38],) and the Felt Arousal Scale (FAS [75],). These scales correspond to the core affective dimensions of valence and activation, respectively. The FS is an 11-point scale ranging from -5 (“Very bad”) to + 5 (“Very good”), and the FAS is a 6-point scale ranging from 1 (“Low arousal”) to 6 (“High arousal”). FS and FAS data collected at six time points were used for analyses in accordance with recommendations for different exercise modalities [5, 41]. The specific time points were: the 10th and 15th min of the aerobic activity during the initial stage, the final measurement associated with the lat pulldown, the 5th and 15th min of the aerobic activity during the fundamental phase, and the last measurement taken during the stretching activities.

Post-exercise enjoyment and core affective exercise experiences were designated as secondary outcomes of interest, with the scores per group compared from pre- to post-intervention. Enjoyment was measured with an eight-item (e.g., “It’s no fun at all”) instrument answered in a 7-point Likert scale ranging from 1 (“Totally disagree”) to 7 (“Totally agree”). Regarding core affective exercise experiences, the pleasure-displeasure factor of the Affective Exercise Experiences (AFFEXX) questionnaire [26] was used. This factor is composed of four items accompanied by a 7-point Likert scale with antithetically worded statements (e.g., “exercise makes me feel worse – exercise makes me feel better”). The AFFEXX was translated to Portuguese in accordance with recommendations (e.g., [9, 10]).

Finally, anticipated and recalled affective responses were measured at all sessions and analyzed between groups and across sessions. Anticipated affective responses were assessed with the Empirical Valence Scale (EVS; [53]), adapted as suggested in a related study (Zenko et al., 2016 [87]). This instrument uses 15 empirically spaced verbal anchors ranging from -100 (“Most unpleasant imaginable”) to + 100 (“Most pleasant imaginable”). The EVS was administered immediately before each session, following a brief explanation of what activities were to be performed during that session, with the instruction, “Considering the workout you are about to begin, how do you think it will make you feel?”. As for remembered affect, a Visual Analog Scale (VAS) ranging from + 100 (“Very pleasant”) to -100 (“Very unpleasant”) was administered a few minutes before the sessions began, starting with the second session (i.e., “How did your last workout make you feel?”). More information regarding the study instruments can be found in the trial protocol [77].

### Manipulation checks

Heart rate was monitored as a manipulation check through the continuous use of a Polar H10 (Polar Electro Inc., Kempele, Finland). This instrument was paired with the Elite HRV app (Version: 5.5.4; Gloucester, Massachusetts), validated for field testing [62]. Blood pressure was measured during the pre-intervention evaluation session using an OMRON M3 Intellisense HEM7051-E instrument (OMRON Healthcare Europe, B.V., Hoofddorp, The Netherlands). Sleep, stress, fatigue, and muscle soreness were measured through the Hooper questionnaire [43]. Participants rated their perceptions on a scale ranging from 1 to 7. For sleep, verbal descriptors ranged from “Very, very good” to “Very, very bad”; for stress, fatigue, and delayed-onset muscle soreness, the anchors ranged from “Very, very low” to “Very, very high”. The sum of all categories is represented as the Hooper index.

No changes in trial outcomes from the published study protocol occurred. However, due to space constraints, one secondary hypothesis specified in the trial protocol (regarding the relation of the slope of affect, end-session affect, and affective peaks with anticipated and remembered affect) will be presented in a separate report.

### Sample size

The sample size was calculated a priori using G*Power (v.3.1.9.7; [30]). The planned analysis for the primary outcome involved a split-plot ANOVA (2 groups × 8 time points). Anticipating an effect size *f* = 0.20 for the interaction, with an α = 0.05, statistical power 1 – β = 0.90, a correlation between the repeated measurements of *r* = 0.70, and a violation of sphericity (ε = 0.70), a total sample size of 24 individuals (12 in each group) was needed. Sample size calculations also took into account the secondary outcomes. When considering the most restrictive analysis to be performed (a split-plot ANOVA [2 groups × 2 time points]), the sample size needed was 42 individuals (21 per group). With an oversampling of 10% to account for attrition, a total of ~ 46 individuals (23 in each group) was specified as the recruitment target.

### Randomization

#### Sequence generation, allocation, and blinding

Given the rolling-recruitment nature of the study, a simple randomization procedure was applied. Randomization took place after baseline data collection and immediately before the first session. The only exception was the last participant to be recruited, who was allocated to the group with one remaining open spot, to ensure an equal number of participants per group. This was a single-blinded trial. As noted, participants were unaware of the group to which they were randomized or the nature of the interventions being compared in the trial. Researchers AJA and VB performed the randomization. Only the two researchers (AJA and VB) implementing the exercise interventions were (unavoidably) aware of the group allocation for each participant. The researchers involved in data screening and statistical analysis had no contact with the participants and remained blinded to group allocation.

### Statistical methods

After initial data screening, descriptive (means and standard deviations), normality (skewness between -2 and 2, kurtosis between -7 and 7), and homoscedasticity (Box’s and Levene’s tests of equality of variances) analyses were conducted. Pre-to-post differences (*Δ*) were calculated by subtracting the average scores of the experimental group from the control group. Independent t-tests and Chi-square test were performed for baseline group comparisons with *p* < 0.05 set for significance analysis. For comparisons between means, Cohen’s *d* was calculated, and the values were characterized as indicating a “small” effect (*d* = 0.20), “medium” effect (*d* = 0.50), and “large” effect (*d* = 0.80), as per recommendations [13], p. 48).

The primary outcome (i.e., the difference between groups in weekly exercise frequency post-intervention) was examined through a split-plot ANOVA (2 groups × 8 time points). Secondary outcomes were assessed through split-plot ANOVAs according to the number of variables to be tested (2 groups x number of time points), as defined in the trial protocol [77]. If violations of sphericity were detected (i.e., for analyses involving more than two time points for the within-subjects factor), the Greenhouse–Geisser adjustment was applied to the degrees of freedom. For significant main effects and interactions, pairwise comparisons (Bonferroni-corrected for the main effects and Fisher’s Least Significant Difference for the interactions) were performed. The uncorrected *p* values from the pairwise comparisons were multiplied by the number of comparisons, thus allowing the application of the customary criterion of *p* < 0.05. For main effects and interactions, partial eta-square (η^2^_p_) effect sizes were calculated and used for the interpretation, according to the benchmarks proposed by Cohen [13], namely “small” (0.01), “medium” (0.06), and “large” (0.14). Mean differences were calculated, and the respective 95% CI are presented.

## Results

**Fig. 1:**
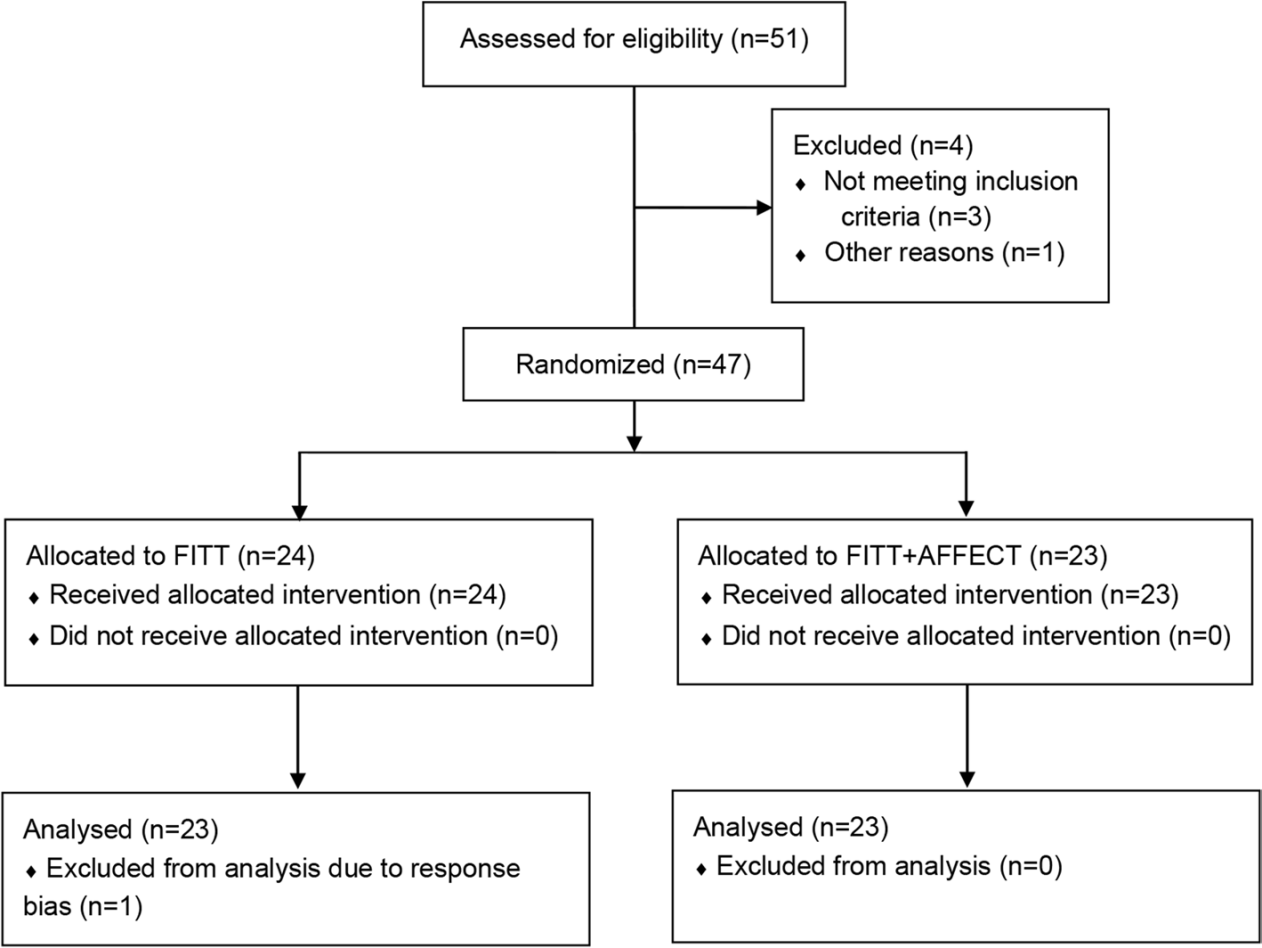
CONSORT flow diagram

Fifty-one participants were assessed for eligibility. Of these, four were excluded, three due to not meeting the inclusion criteria (blood pressure ≥ 130/80 mmHg, *n* = 2; BMI > 29.9 kg/m^2^; *n* = 1) and one because of an artificial hip joint, possibly masking bodily sensations (*n* = 1). Forty-seven participants were randomly assigned to the control (FITT; *n* = 24) and experimental (FITT + AFFECT; *n* = 23) groups. All allocated participants received the intended interventions. However, a decision was made during the intervention (i.e., before the completion of data collection) that the data from one participant in the control group had to be discarded due to obvious response bias (i.e., extreme social desirability bias and/or random responses). As a result, we oversampled by one (hence, 24 participants in total were allocated to the control group) to compensate. The recruitment process took approximately four months, and the follow-up period was eight weeks post-intervention. All data were collected before the end of January 2023. Details of the participant flow can be found in Fig. 1.

Descriptive and inferential analyses were preceded by initial screening and testing for assumptions. Sample baseline characteristics are presented in Table 1. The groups exhibited only minor differences in age, BMI, and exercise frequency. However, the control group presented some imbalance in the proportion of men and women.^1^

**Table 1:** Descriptive statistics and group differences of sample baseline characteristics

	Total sample (*n* = 46)		Control group (*n* = 23)		Experimental group (*n* = 23)	
	*M*	*SD*	*M*	*SD*	*M*	*SD*
Age (years)	32.00	8.62	32.26	7.82	31.74	9.52
BMI (kg/m^2^)	24.34	2.72	23.74	2.70	24.94	2.67
Six-month pre-intervention exercise frequency (sessions per week)	0.59	0.45	0.46	0.47	0.72	0.41
	Male, *n*	Female, *n*	Male, *n*	Female, *n*	Male, *n*	Female, *n*
Sex	20 (43.5%)	26 (56.5%)	8 (34.8%)	15 (65.2%)	12 (52.2%)	11 (47.8%)

*Note*. *n* = sample size, *M* = mean, *SD* = standard deviation

Resistance training volume, average session heart rate, and fatigue index scores were collected for descriptive purposes and are presented in Table 2. The average heart rate was within the moderate-intensity range for both groups (68% and 66% of age-predicted maximal heart rate in the control and experimental group, respectively). Resistance training volume increased across the three intervention sessions in both groups, with the experimental group presenting higher volumes (from 6,701 to 8,165 kg in the control group, from 7,289 to 8,854 in the experimental group). Fatigue indicators through the Hooper index exhibited decreasing values across sessions for both groups and all were below the scale midpoint of 14 points (from 10.00 to 9.65 in the control group, from 11.00 to 9.48 in the experimental group). No significant differences were found between groups in each session.

**Table 2:** Descriptive statistics, group differences, and effect sizes for the total volume of resistance training, session heart rate, and fatigue indicators across the three individualized sessions

	Control group (*n* = 23)			Experimental group (*n* = 23)						
	*M*	*SD*	(%Max HR)	*M*	*SD*	(%Max HR)	*t*	*p*	*Δ*	*d*
Heart rate (bpm) session 1	124.98	9.66	(67.4%)	124.89	17.17	(67.2%)	0.02	.982	0.10	0.01
Heart rate (bpm) session 2	127.79	11.23	(68.9%)	123.95	14.53	(66.7%)	1.00	.321	3.84	0.30
Heart rate (bpm) session 3	125.65	11.22	(67.8%)	121.19	13.20	(65.2%)	1.23	.224	4.45	0.36
	*M*	*SD*		*M*	*SD*		*t*	*p*	*Δ*	*d*
Total volume (kg) session 1	6701	2047		7289	2918		-0.79	.433	-588.00	-0.23
Total volume (kg) session 2	7813	2245		8160	3328		-0.41	.681	-346.96	-0.12
Total volume (kg) session 3	8165	2446		8854	3635		-0.75	.455	-688.69	-0.22
Hooper index session 1	10.00	2.98		11.00	3.26		-1.09	.284	-1.00	-0.32
Hooper index session 2	9.78	2.92		10.04	3.07		-0.30	.769	-0.26	-0.09
Hooper index session 3	9.65	2.98		9.48	3.44		0.18	.855	0.17	0.05

*Note*. *n* = sample size, kg = kilograms, bpm = beats per min, *M* = mean, *SD* = standard deviation, %Max HR = Percentage of age-predicted maximum heart rate [76], *t* = independent *t*-test, *p* = significance, Δ = difference between the control group and the experimental group, *d* = Cohen’s effect size

### Primary outcome

**Fig. 2:**
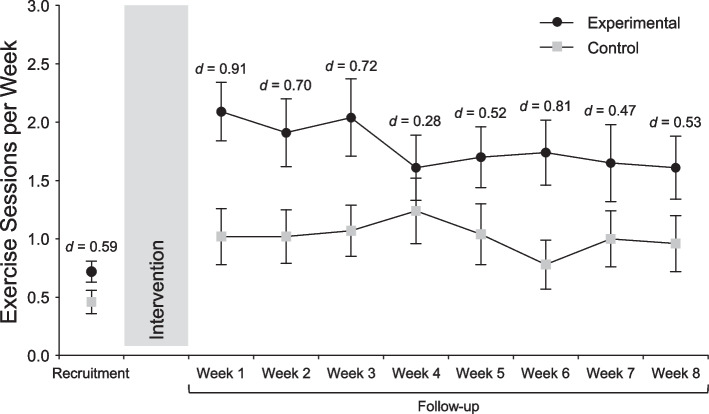
Mean (± SE) weekly exercise session attendance in the experimental and control groups during the eight-week follow-up period

Over the eight-week follow-up period, participants in the control group visited the gymnasia an average of 8.13 times, whereas participants in the experimental group averaged 14.35 visits, a difference of 77%. As shown in Table 3 and Fig. 2, the ANOVA on weekly exercise frequency over the eight-week follow-up period showed only a significant main effect of Group, *F* (1, 44) = 6.010, *p* = 0.018; η^2^_p_ = 0.120. This indicates that, across the entire follow-up period, the exercise frequency of the experimental group was significantly higher than that of the control group, with a difference that approached a “large” effect.

**Table 3:** Descriptive statistics and split-plot ANOVA results for the study's primary outcome (exercise frequency during the eight weeks post-intervention)

	Control group (*n* = 23)		Experimental group (*n* = 23)			Time main effect			Group by Time interaction			Group main effect			Mean difference (95% CI)		
	*M*	*SD*	*M*	*SD*	*d*	*F*	*p*	η^2^_p_	*F*	*p*	η^2^_p_	*F*	*p*	η^2^_p_	*Δ*	LBCI	UBCI
Week 1 frequency	1.02	1.15	2.09	1.20	-0.91	1.138	.341	.025	1.172	.319	.026	6.010	.018	.120	-1.065	-1.765	-0.365
Week 2 frequency	1.02	1.12	1.91	1.41	-0.70										-0.896	-1.652	-0.140
Week 3 frequency	1.07	1.05	2.04	1.58	-0.72										-0.974	-1.770	-0.178
Week 4 frequency	1.24	1.33	1.61	1.34	-0.28										-0.370	-1.163	0.424
Week 5 frequency	1.04	1.26	1.70	1.26	-0.52										-0.652	-1.401	0.096
Week 6 frequency	0.78	0.99	1.74	1.36	-0.81										-0.957	-1.664	-0.249
Week 7 frequency	1.00	1.17	1.65	1.56	-0.47										-0.652	-1.469	0.165
Week 8 frequency	0.96	1.15	1.61	1.31	-0.53										-0.652	-1.382	0.078
Sum exercise sessions	8.13	-	14.35	-											-	-	-

*Note. n* = sample size, *M* = mean, *SD* = standard deviation, *d* = Cohen's effect size,* F* = value of the *F* ratio, *p* = probability of *F*, η^2^_p_ = partial eta-squared effect size, *CI* = confidence interval, *LBCI* = lower bound of the 95% confidence interval, *UBCI* upper bound of the 95% confidence interval

On the other hand, there was neither a main effect of Time (*p* = 0.341, η^2^_p_ = 0.025) nor a Group-by-Time interaction (*p* = 0.319, η^2^_p_ = 0.026). These results indicate that the difference between the two groups did not diminish over the follow-up period.

### Secondary outcomes

**Fig. 3:**
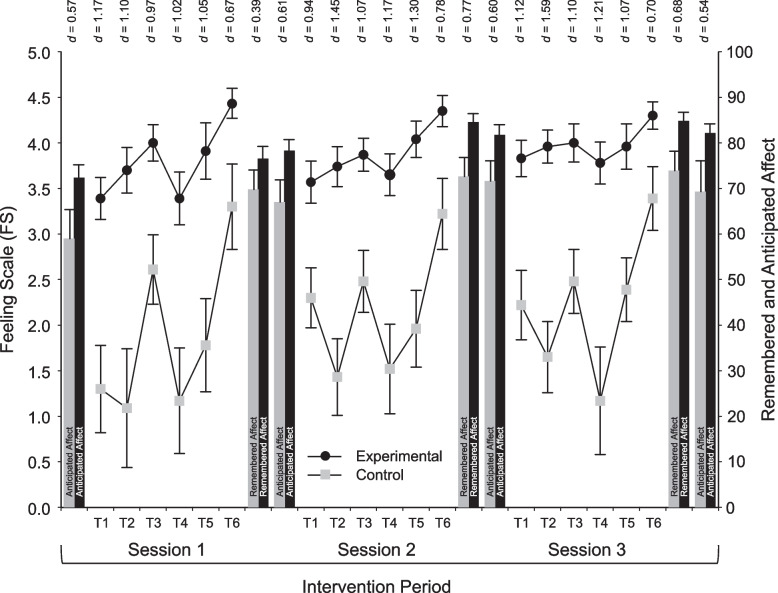
Means (± SE) of the Experimental and Control groups on the Feeling Scale (left vertical axis), Remembered Affect, and Anticipated Affect (right vertical axis) across the three intervention sessions. *Note*. The six time points during each session represent: (T1) 10th min of aerobic activity (initial stage), (T2) 15th min of aerobic activity (initial stage), (T3) final measurement of lat pulldown, (T4) 5th min of aerobic activity (fundamental phase), (T5) 15th min of aerobic activity (fundamental phase), and (T6) last measurement during stretching

**Fig. 4:**
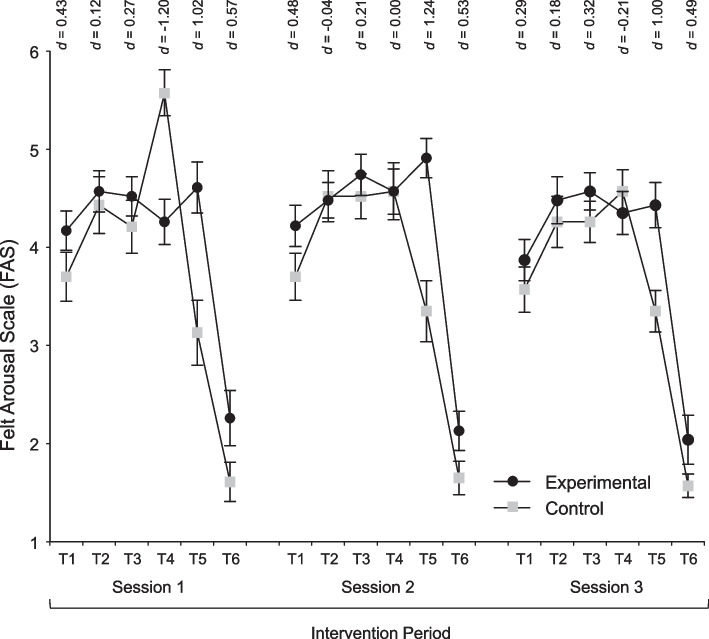
Means (± SE) of the Experimental and Control groups on the Felt Arousal Scale (FAS) across the three intervention sessions. *Note.* The six time points during each session represent: (T1) 10th min of aerobic activity (initial stage), (T2) 15th min of aerobic activity (initial stage), (T3) final measurement of lat pulldown, (T4) 5th min of aerobic activity (fundamental phase), (T5) 15th min of aerobic activity (fundamental phase), and (T6) last measurement during stretching

Results pertaining to FS and FAS are presented in Table 4, and Figs. 3 and 4. The experimental manipulation produced ratings of affective valence that, averaged across each session, were higher in the experimental than the control group by 1.93, 1.72, and 1.76 units on the 11-point FS during the first, second, and third intervention session, respectively. The analysis of FS ratings (also see Fig. 3) revealed that, for each intervention session, there were significant and large main effects of Time (*p* < 0.001) and significant and large main effects of Group (*p* < 0.001), as well as a significant, medium-sized Group-by-Time interaction only for Session 3, *F* = 3.77, *p* = 0.007, η^2^_p_ = 0.080. This indicates that, for each intervention session, despite oscillations in affective valence across the phases of each session, the experimental manipulation was highly effective in creating more pleasant exercise experiences compared to the comparator (i.e., standard) exercise prescription.

**Table 4:** Descriptive statistics and split-plot ANOVA results for the study's secondary outcomes (during exercise; affective responses)

		Control group (*n* = 23)		Experimental group (*n* = 23)			Time main effect			Group by Time interaction			Group main effect			Mean difference (95% CI)		
		*M*	*SD*	*M*	*SD*	*d*	*F*	*p*	η^2^_p_	*F*	*p*	η^2^_p_	*F*	*p*	η^2^_p_	*Δ*	LBCI	UBCI
Session 1	Feeling Scale timepoint 1	1.30	2.29	3.39	1.08	-1.17	8.49	< .001	.16	1.61	.186	.04	21.96	< .001	.33	-2.090	-3.148	-1.026
	Feeling Scale timepoint 2	1.09	3.13	3.70	1.22	-1.10										-2.610	-4.022	-1.196
	Feeling Scale timepoint 3	2.61	1.80	4.00	0.95	-0.97										-1.390	-2.248	-0.534
	Feeling Scale timepoint 4	1.17	2.77	3.39	1.37	-1.02										-2.220	-3.518	-0.917
	Feeling Scale timepoint 5	1.78	2.43	3.91	1.50	-1.05										-2.130	-3.332	-0.929
	Feeling Scale timepoint 6	3.30	2.26	4.43	0.79	-0.67										-1.130	-2.138	-0.123
	Felt Arousal Scale timepoint 1	3.70	1.22	4.17	0.94	-0.43	46.83	< .001	.52	4.48	.005	.09	3.66	.062	.08	-0.470	-1.125	0.169
	Felt Arousal Scale timepoint 2	4.43	1.38	4.57	0.99	-0.12										-0.140	-0.843	0.582
	Felt Arousal Scale timepoint 3	4.21	1.31	4.52	0.95	-0.27										-0.310	-0.985	0.376
	Felt Arousal Scale timepoint 4	5.57	1.08	4.26	1.10	1.20										1.310	-0.342	0.951
	Felt Arousal Scale timepoint 5	3.13	1.60	4.61	1.27	-1.02										-1.480	-2.338	-0.618
	Felt Arousal Scale timepoint 6	1.61	0.94	2.26	1.32	-0.57										-0.650	-1.334	0.030
Session 2	Feeling Scale timepoint 1	2.30	1.58	3.57	1.08	-0.94	7.36	< .001	.14	2.44	.054	.05	25.78	< .001	.37	-1.261	-2.065	-0.457
	Feeling Scale timepoint 2	1.43	2.00	3.74	1.05	-1.45										-2.304	-3.253	-1.356
	Feeling Scale timepoint 3	2.48	1.62	3.87	0.87	-1.07										-1.391	-2.164	-0.619
	Feeling Scale timepoint 4	1.52	2.33	3.65	1.11	-1.17										-2.130	-3.217	-1.044
	Feeling Scale timepoint 5	1.96	2.03	4.04	0.98	-1.30										-2.087	-3.035	-1.139
	Feeling Scale timepoint 6	3.22	1.88	4.35	0.83	-0.78										-1.130	-1.995	-0.266
	Felt Arousal Scale timepoint 1	3.70	1.15	4.22	1.00	-0.48	65.25	< .001	.60	5.27	.001	.11	4.09	.049	.09	-0.522	-1.16	0.117
	Felt Arousal Scale timepoint 2	4.52	1.24	4.48	0.85	0.04										0.043	-0.587	0.674
	Felt Arousal Scale timepoint 3	4.52	1.08	4.74	1.01	-0.21										-0.217	-0.839	0.404
	Felt Arousal Scale timepoint 4	4.57	1.38	4.57	1.12	0.00										0.001	-0.746	0.746
	Felt Arousal Scale timepoint 5	3.35	1.50	4.91	0.95	-1.24										-1.565	-2.310	-0.821
	Felt Arousal Scale timepoint 6	1.65	0.83	2.13	0.97	-0.53										-0.478	-1.015	0.058
Session 3	Feeling Scale timepoint 1	2.22	1.81	3.83	0.94	-1.12	9.67	< .001	.17	3.77	.007	.08	23.90	< .001	.35	-1.609	-2.464	-0.753
	Feeling Scale timepoint 2	1.65	1.85	3.96	0.88	-1.59										-2.304	-3.165	-1.444
	Feeling Scale timepoint 3	2.48	1.68	4.00	1.00	-1.10										-1.522	-2.342	-0.702
	Feeling Scale timepoint 4	1.17	2.84	3.78	1.09	-1.21										-2.609	-3.886	-1.331
	Feeling Scale timepoint 5	2.39	1.70	3.96	1.19	-1.07										-1.565	-2.436	-0.695
	Feeling Scale timepoint 6	3.39	1.70	4.30	0.70	-0.70										-0.913	-1.686	-0.141
	Felt Arousal Scale timepoint 1	3.57	1.08	3.87	1.01	-0.29	66.28	< .001	.60	2.92	.029	.06	2.92	.094	.06	-0.304	-0.927	0.318
	Felt Arousal Scale timepoint 2	4.26	1.25	4.48	1.16	-0.18										-0.217	-0.935	0.500
	Felt Arousal Scale timepoint 3	4.26	1.01	4.57	0.90	-0.32										-0.304	-0.872	0.263
	Felt Arousal Scale timepoint 4	4.57	1.04	4.35	1.07	0.21										0.217	-0.409	0.844
	Felt Arousal Scale timepoint 5	3.35	1.03	4.43	1.12	-1.00										-1.087	-1.726	-0.448
	Felt Arousal Scale timepoint 6	1.57	0.59	2.04	1.22	-0.49										-0.478	-1.049	0.093

*Note. n* = sample size, *M* = mean, *SD* = standard deviation, *d* = Cohen's effect size,* F* = value of the *F* ratio, *p* = probability of *F*, η^2^_p_ = partial eta-squared effect size, *CI* confidence interval, *LBCI *lower bound of the 95% confidence interval, *UBCI* upper bound of the 95% confidence interval

Consistent with the goal of the intervention, which was to improve affective valence rather than perceived activation, the intergroup differences in ratings of perceived activation, averaged across each session, remained below one-half unit on the 6-point FAS (0.29, 0.46, 0.36 higher in the experimental than the control group during the first, second, and third session, respectively). The analysis of FAS ratings (also see Fig. 4) showed, across all three intervention sessions, significant and large effects of Time (*p* < 0.001), as well as significant and medium-sized Group-by-Time interactions (η^2^_p_ from 0.06 to 0.11). The Group main effect reached significance (*p* = 0.049, η^2^_p_ = 0.090) only for Session 2 (the experimental group scored higher than the control group).

As can be seen in Table 5, there were no significant results for post-exercise enjoyment (assessed following each intervention session) and affective exercise experiences (assessed at baseline and at the conclusion of the trial). The Group main effect, Time main effect, and Group-by-Time interaction were all non-significant (*p* > 0.05).

**Table 5:** Descriptive statistics and split-plot ANOVA results for the secondary outcomes (enjoyment, core affective experiences, remembered affect, and anticipated affect)

	Control group (*n* = 23)		Experimental group (*n* = 23)			Time main effect			Group by Time interaction			Group main effect			Mean difference (95% CI)		
	*M*	*SD*	*M*	*SD*	*d*	*F*	*p*	η^2^p	*F*	*p*	η^2^p	*F*	*p*	η^2^p	*Δ*	LBCI	UBCI
Enjoyment Session 1	5.64	1.18	5.92	0.64	-0.29	.026	.918	.001	.439	.557	.010	1.426	.239	.031	-0.280	-0.840	0.286
Enjoyment Session 2	5.59	1.28	5.96	0.71	-0.36										-0.370	-0.990	0.240
Enjoyment Session 3	5.56	1.41	5.97	0.77	-0.36										-0.410	-1.083	0.268
Core affective exercise experiences baseline	6.03	1.14	6.24	0.50	-0.07	.664	.420	.015	.005	.941	.000	.836	.366	.019	-0.207	-0.729	0.316
Core affective exercise experiences post-trial	6.10	0.84	6.29	0.48	-0.10										-0.196	-0.601	0.209
Remembered affect 1	69.78	20.48	76.52	13.00	-0.39	4.983	.012	.102	.827	.428	.018	5.775	.021	.116	-6.740	-16.933	3.455
Remembered affect 2	72.61	20.22	84.57	8.91	-0.77										-11.960	-21.241	-2.672
Remembered affect 3	73.91	20.45	84.78	9.35	-0.68										-10.870	-20.318	-1.421
Anticipated affective response 1	59.00	30.46	72.39	13.30	-0.57	5.685	.004	.110	.410	.886	.010	5.59	.022	.114	-13.390	-27.357	0.575
Anticipated affective response 2	67.04	23.33	78.26	11.74	-0.61										-11.220	-22.194	-0.241
Anticipated affective response 3	71.61	21.43	81.74	10.72	-0.60										-10.130	-20.202	-0.059
Anticipated affective response 4	69.30	32.40	82.17	9.75	-0.54										-12.870	-27.088	1.349

*Note. n* = sample size, *M* = mean, *SD* = standard deviation, *d* = Cohen's effect size,* F* = value of the *F* ratio, *p* = probability of *F*, η^2^_p_ = partial eta-squared effect size, *CI *confidence interval, *LBCI* lower bound of the 95% confidence interval, *UBCI* upper bound of the 95% confidence interval

The ANOVAs on remembered and anticipated affect showed a similar pattern of results (see Table 5). For both variables, the Group main effect and the Time main effect were significant, with effect sizes between medium and large (η^2^_p_ from 0.102 to 0.116). On the other hand, neither Group-by-Time interaction was significant. The Time main effect indicates that both variables exhibited an upward trend across sessions. Specifically, remembered affect increased from a low of 69.78 to a high of 73.91 in the control group and from a low of 76.52 to a high of 84.78 in the experimental group. Likewise, anticipated affect increased from a low of 59.00 to a high of 71.61 in the control group and from a low of 72.39 to a high of 82.17 in the experimental group. However, in each case, even the highest rating achieved by the control group was still lower than the lowest rating achieved by the experimental group, which accounted for the significant Group main effect. No harms were detected during the session or in the follow-up period.

## Discussion

The present intervention was translational in nature. The idea of encouraging the self-regulation of exercise intensity and, more specifically, instructing participants to use affect as a basis of self-regulating exercise intensity, with the goal of improving affective responses had received considerable empirical support from laboratory-based studies [2, 19, 49, 58, 81]. This RCT tested these methods in the pragmatic context of commercial gymnasia and assessed their impact on exercise behavior using an objective measure of session attendance. The results were in line with expectations and demonstrate the practicality and effectiveness of instructions focused on the self-regulation of exercise intensity as a method of enhancing the affective experience of exercise and, in turn, increasing exercise behavior. Specifically, in the present study, the experimental manipulation resulted in average intergroup differences between 1.5 and 2.0 units on the FS during the three intervention sessions, respectively. In turn, over the eight weeks of follow-up the two groups exhibited a difference in session attendance of 77%. This pattern is consistent with the results of previous studies, which had demonstrated that a one-unit improvement in FS ratings during exercise was associated with 27–29 additional min of physical activity per week cross-sectionally and an increase of 15 min [84] to 38 min [82] per week 6 months later, and 41 min 12 months later [82].

Importantly, intergroup differences in gymnasium attendance in the present trial did not appear to diminish over the course of the eight-week follow-up period. With the exception of the fourth week, at which point the effect size was small, intergroup differences ranged between large to medium. This indicates that, while the longer-term sustainability of these effects remains to be established, the cost–benefit proposition should be appealing to both exercise professionals and participants. Without requiring technical or technological means (e.g., audiovisual or computer equipment, as in the case of music, video, or virtual-reality interventions) and without necessitating prior practice (as in the case of cognitive reappraisal interventions), participants derived meaningful benefits simply as a result of receiving modified instructions about how to self-regulate their intensity, in a brief intervention period.

It could be argued that the greatest difficulty in implementing the intervention that was tested in the present study might be convincing exercise professionals to think beyond the long-entrenched FITT principle, and to prioritize sustained participation as a prerequisite for attaining fitness and health benefits. Specifically, one possible perceived barrier for exercise professionals in implementing the method of self-selection of intensity or the affect-guided self-regulation of intensity is the suspicion that, when given autonomy, many or most individuals would choose a low level of intensity, thus depriving themselves of potential fitness and health benefits. It is, therefore, important to underscore that, in line with previous evidence (see [19], for a review), intergroup differences in the session-average heart rate and the total volume of resistance exercise were small, with the former slightly in favor of the control group but the latter in favor of the experimental group. These results align with those from an acute study in the context of school physical education, in which an innovative lesson designed to improve affective responses, in part by promoting autonomy, improved affect, and enjoyment but did not differ in terms of accelerometry-assessed moderate-to-vigorous physical activity from a “traditional” physical education lesson [80].

Examined from a broader perspective, the results of the present study have implications for the assumptions underpinning the development of exercise prescription guidelines. The widely used guidelines by the American College of Sports Medicine [1] emphasize the importance of individualization for all modalities of exercise, including aerobic and resistance activities. However, the main purpose of individualization is portrayed as the minimization of the risk of adverse physical and physiological occurrences, such as symptoms of hypoperfusion, muscular and skeletal injury, muscle soreness, undue fatigue, and overtraining. What is still missing is the full integration of psychological considerations into the exercise prescription guidelines. For example, the guidelines make passing reference to the need for progression of the “dose” to be “individualized and tailored to tolerance and preference” (p. 185). However, how individualization and tailoring should be implemented is left unspecified. Interventions aimed at encouraging adherence appear in the guidelines as a process that should unfold in parallel to, rather than fully integrated with, the exercise prescription.

The present results add to a voluminous research literature [23, 24] demonstrating that the continued lack of integration of psychological considerations into exercise prescription guidelines is inconsistent with the extant empirical evidence and, therefore, likely detracts from the goal of promoting exercise and physical activity participation and maintenance. The intervention that was implemented in the present RCT incorporated several lessons from lines of evidence that have emerged from exercise-psychology research. Together, these elements compose an alternative, highly practical, easily implementable, and evidence-supported approach to exercise intensity prescription, centered on the notion of individualization that is already endorsed by the American College of Sports Medicine [1]. The approach is readily available to exercise professionals. First, individual differences in preference for and tolerance of exercise intensity were assessed by self-report as part of preparticipation screening [39, 69]. Both constructs have been shown to predict affective responses to exercise, the former more so when the intensity is self-selected or moderate and the latter more so when the intensity is imposed or heavy [69]. Second, the participants were instructed to self-regulate their intensity to promote a pleasant affective experience during the exercise sessions. These instructions have also been shown to be effective in accomplishing the dual goal of improving the affective experience of exercise while, at the same time, preserving its fitness-enhancing and health-promoting potential [4, 15, 28, 37, 56, 57, 66, 86].

It is remarkable that the combination of these two methods, which have been proposed in the exercise-psychology literature for nearly 20 years [22, 66], produced large intergroup differences in ratings of affective valence throughout the three intervention sessions, as well as medium-to-large differences in remembered and anticipated affect. Examination of the patterns of affective responses during the three intervention sessions showed that valence and activation exhibited considerable variation over time, as participants engaged in different modalities of exercise (i.e., aerobic, resistance, stretching). It is noteworthy, for example, that the experimental and control groups had divergent responses to the progressive aerobic activity on a treadmill, with the experimental group exhibiting an improvement in ratings of affective valence between the 10th and 15th min, whereas the control group exhibited declines. At that time, participants progressed from moderate-intensity activity (first 10 min) to vigorous-intensity activity (last five min). As noted in the introduction, an important benefit of instructions that emphasize autonomy, compared to standard instructions in which participants are told to comply with a prescribed intensity target (e.g., a heart rate zone), is that affective responses can “uncouple” from the level that would have been expected from the level of physiological perturbation, thus enabling participants to feel better even while exercising at vigorous intensity [24]. Another important observation is that, although the final period of stretching exercises was reported by both groups as the most pleasant part for all three intervention sessions [41, 42], and the experimental group exhibited signs of a ceiling effect (*M*_*1*_ ± *SD* = 4.43 ± 0.79; *M*_*2*_ ± *SD* = 4.35 ± 0.83; *M*_*3*_ ± *SD* = 4.30 ± 0.70 out of a maximum of 5.00), the intergroup differences consistently approached a “large” effect size (*d*_*1*_ = 0.67; *d*_*2*_ = 0.78; *d*_*3*_ = 0.70) in favor of the experimental group.

It should be noted that the intervention did not produce significant differences in the secondary outcomes of post-exercise enjoyment and the core-affect component of affective exercise experiences [26]. Although not supporting our hypothesis, there may be reasonable explanations for the lack of significant differences. Enjoyment has been shown to be associated with self-reported physical activity [64] and to respond to experimental manipulations [65]. However, enjoyment has an inconsistent association with affective responses and likely reflects a mixture of affective experiences and cognitive appraisals, especially when assessed several minutes after the end of exercise sessions [25]. For example, an individual may experience displeasure during strenuous exercise but may report a high level of enjoyment several minutes after exercise, when homeostatic perturbations have subsided, and the strenuous session has been cognitively reinterpreted as a “success” or as evidence of perseverance in the face of challenge. On the other hand, the construct of “affective exercise experiences” was theorized as an affective concept, but one that is unlikely to be responsive to a short-term intervention because it reflects the totality of affective experiences from exercise over the course of a lifetime: “affective exercise experiences as a summary valenced designation, ranging from pleasant to unpleasant, that reflects the history of associations between exercise over the life course of an individual and the attendant affective responses” [26], pp. 2–3). In the present RCT, the three-session intervention successfully manipulated affective responses, but this was evidently an insufficient stimulus to alter the “summary valenced designation” of exercise.

The main strengths of this RCT include its theory-guided hypothesis, its pragmatic nature, the easily translatable and scalable intervention, the preregistration of its methods (which remained unaltered during implementation), and absence of any detected harms. On the other hand, as is the case with all studies, readers should take into account certain limitations, which can also be construed as directions for future research. First, an important caveat is the short intervention and follow-up periods. From one perspective, demonstrating that three intervention sessions can yield effects that remain meaningful and undiminished over an eight-week follow-up period can be seen as an indication of high efficiency (i.e., a minimal initial investment in terms of cost and time producing benefits still observable after several weeks). At the same time, however, establishing that a sustainable behavior change has taken place requires observations over a longer time scale. In that sense, the present data should be seen as a valuable initial “proof of concept,” but future trials should seek to extend the duration of both the intervention and the follow-up periods.

Second, the non-blocked nature of randomization resulted in almost equal numbers of male and female participants in the experimental group but not so in the control group (34.8% male vs. 65.2% female). Although we have no reason to believe that this imbalance affected the outcomes, it should be taken into consideration.

Third, it is presently unknown how to best represent affective responses obtained over extended and heterogeneous episodes of experience, such as exercise sessions, by a “reduced” number of data points while retaining important information on interindividual variability and intraindividual variation over time. In the present RCT, we had to model exercise sessions taking place in real-life conditions and consisting of diverse modalities (aerobic exercise, resistance exercise, stretching). In cases like this, affective responses clearly cannot be reduced to a linear or quadratic trend over time or a periodic signal that oscillates with a certain frequency. As the present data illustrate, affect responds dynamically to changes in the internal and external environment (e.g., the modality and intensity of exercise). Therefore, we decided to represent the affective experience of each exercise session by six distinct time points we deemed representative on the basis of previous research (see Methods). We made this decision based on prior evidence suggesting that episodes of experience are encoded in memory as more or less pleasant or unpleasant not as a function of the total or the average amount of pleasure experienced during these episodes but rather as a reflection of distinct moments or “snapshots” [32, 46, 47]. The “snapshots” that appear to weigh more heavily in shaping affective memories are those associated with the most pleasant or unpleasant moments, such as the most strenuous part of a workout (e.g., the end of the vigorous aerobic activity) and the moment eliciting the strongest sense of relief or respite (e.g., cool-down and stretching). Conceivably, however, future research may uncover a better approach to represent affective responses associated with complex and heterogeneous episodes of experience.

Fourth, the primary outcome of interest, namely attendance, although directly assessed rather than self-reported, did not provide an indication of the amount of exercise performed per gymnasium visit or the amount of physical activity performed over the follow-up period. The number of visits may not be proportional to the amount of exercise a participant performs per visit or the amount of physical activity an individual does in general (i.e., both inside and outside the gymnasium).

Fifth, future studies should investigate how effective the intervention tested here would be in unsupervised conditions (e.g., in home-based exercise programs). It is conceivable that effectiveness depends on the social dynamics that emerge with the trainer or exercise leader (e.g., perceived accountability or self-fulfilling prophecy) or that the exercise professional is a crucial factor in successful implementation.

Finally, the size of the sample in this study was deemed adequate on the basis of considerations pertaining to statistical significance (i.e., statistical power to enable the testing of a specific hypothesis). However, the adequacy of the sample may also be judged by other considerations (e.g., precision in estimating population values, representativeness and generalizability to the target population; see [51]). For example, larger samples are likely to reflect more heterogeneity or encompass more of the diversity found in populations. Pursuing a larger sample size in future studies would help address these additional considerations and would, therefore, be desirable.

## Conclusion

In conclusion, we showed that, in a pragmatic RCT, three intervention sessions emphasizing individualization and the promotion of pleasure as a guide in self-regulating exercise intensity led to medium-to-large differences in gymnasium attendance that remained undiminished over an eight-week period of follow-up. The brevity and simplicity of the intervention (requiring no technical or technological means and no prior practice) present a readily translatable, scalable, and evidence-supported option for exercise professionals and participants.

## Supplementary Information

Supplementary Material 1.

Supplementary Material 2.

## Data Availability

The datasets used and/or analyzed during the current study are available from the corresponding author upon reasonable request.

## Footnotes

1 Preliminary analyses including sex as a covariate (to investigate the role of sex in an exploratory or hypothesis-generating manner) did not show that sex accounted for significant variance in any of the outcomes. Therefore, sex was not included as a covariate in any of the analyses reported here, in accordance with the trial protocol.
