# Correction: Individualized pleasure‑oriented exercise sessions, exercise frequency, and affective outcomes: a pragmatic randomized controlled trial

**DOI:** 10.1186/s12966-024-01665-9

**Published:** 2024-10-04

**Authors:** Diogo S. Teixeira, Vasco Bastos, Ana J. Andrade, António L. Palmeira, Panteleimon Ekkekakis

**Affiliations:** 1 Faculty of Physical Education and Sport, Lusofona University, Lisbon, Portugal; 2 Research Center in Sport, Physical Education, and Exercise and Health (CIDEFES), Lisbon, Portugal; 3 Michigan State University, Michigan, USA

**Correction** **:** **Int J Behav Nutr Phys Act 21, 85 (2024)**

**https://doi.org/10.1186/s12966-024-01636-0**

Following the publication of the Original Article, the authors reported some errors.

With regards to Table 3, some data were incorrectly transferred. This resulted to misaligned data when the control to the experimental group mean frequency per week are subtracted.

**Incorrect**

**Table:** 

		Mean difference (95% CI)		
		*Δ*	LBCI	UBCI
Week 1 frequency	…	-0.875	-1.607	-0.143
Week 2 frequency		-0.875	-1.629	-0.121
Week 3 frequency		-0.962	-1.757	-0.167
Week 4 frequency		-0.353	-1.144	0.438
Week 5 frequency		-0.614	-1.357	0.129
Week 6 frequency		-0.918	-1.599	-0.238
Week 7 frequency		-0.658	-1.428	0.112
Week 8 frequency		-0.614	-1.339	0.111
Sum exercise sessions		-	-	-

**Correct**

**Table:** 

		Mean difference (95% CI)		
		*Δ*	LBCI	UBCI
Week 1 frequency	…	**-1.065**	**-1.765**	**-0.365**
Week 2 frequency		**-0.896**	**-1.652**	**-0.140**
Week 3 frequency		**-0.974**	**-1.770**	**-0.178**
Week 4 frequency		**-0.370**	**-1.163**	**0.424**
Week 5 frequency		**-0.652**	**-1.401**	**0.096**
Week 6 frequency		**-0.957**	**-1.664**	**-0.249**
Week 7 frequency		**-0.652**	**-1.469**	**0.165**
Week 8 frequency		**-0.652**	**-1.382**	**0.078**
Sum exercise sessions		-	-	-

With regards to Table 5, some scores were also incorrectly transferred. However, the ANOVA results are presented correctly. These corrections do not affect the results or conclusions of the study.

**Incorrect**

Table 5—Descriptive statistics and split-plot ANOVA results for the secondary outcomes (enjoyment, core affective experiences, remembered affect, and anticipated affect)

**Table:**
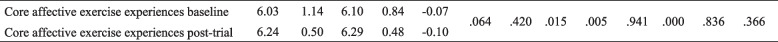

**Correct**

**Table:**
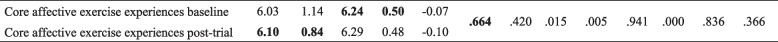

Furthermore, to maintain consistency with the paper's format, all scores should include a zero before the decimal point/comma. The necessary changes have been made to the Results section of the Abstract, as well as to Table 4 and Table 5.

The Original Article has been corrected.
