# Supplementary material for: Individualized pleasure-oriented exercise sessions, exercise frequency, and affective outcomes: a pragmatic randomized controlled trial
Source: Int J Behav Nutr Phys Act. 2024 Aug 5;21:85. doi: 10.1186/s12966-024-01636-0 (PMC11299270; doi:10.1186/s12966-024-01636-0)
Supplement: Supplementary file 1 — Supplementary Material 1. [file 12966_2024_1636_MOESM1_ESM.doc]

Supplementary File 1 - CONSORT 2010 and 2022 extension checklist of information

| Section/Topic | Item No | Checklist item | Reported on page No |
| --- | --- | --- | --- |
| Title and abstract | | | |
|  | 1a | Identification as a randomised trial in the title | Title page, 3 |
|  | 1b | Structured summary of trial design, methods, results, and conclusions (for specific guidance see CONSORT for abstracts) | 1, 2 |
| Introduction | | | |
| Background and objectives | 2a | Scientific background and explanation of rationale | 3-10 |
|  | 2b | Specific objectives or hypotheses | 10 |
| Methods | | | |
| Trial design | 3a | Description of trial design (such as parallel, factorial) including allocation ratio | 10 |
|  | 3b | Important changes to methods after trial commencement (such as eligibility criteria), with reasons | 11 |
| Participants | 4a | Eligibility criteria for participants | 11 |
|  | 4b | Settings and locations where the data were collected | 11 |
| Interventions | 5 | The interventions for each group with sufficient details to allow replication, including how and when they were actually administered | 11-13 |
| Outcomes | 6a | Completely defined pre-specified primary and secondary outcome measures, including how and when they were assessed | 13-15 |
|  | 6a1 | Provide a rationale for the selection of the domain for the trial’s primary outcome | 3-10 |
|  | 6a2 | Describe the specific measurement variable (eg, systolic blood pressure), analysis metric (eg, change  from baseline, final value, time to event), method of aggregation (eg, mean, proportion), and the time point  for each outcome | 13-16 |
|  | 6a3 | If the analysis metric for the primary outcome represents within-participant change, define and justify the minimal important change in individuals | NA |
|  | 6a4 | If the outcome data were continuous, but were analyzed as categorical (method of aggregation), specify the cutoff values used | NA |
|  | 6a5 | If outcome assessments were performed at several time points after randomization, state the time points used for the analysis | NA |
|  | 6a6 | If a composite outcome was used, define all individual components of the composite outcome | NA |
|  | 6a7 | Identify any outcomes that were not prespecified in a trial registry or trial protocol | NA |
|  | 6a8 | Provide a description of the study instruments used to assess the outcome (eg, questionnaires, laboratory tests) along with reliability, validity, and responsiveness in a population similar to the study sample | 14-15 |
|  | 6a9 | Describe who assessed the outcome (eg, nurse, parent) and any qualifications or trial-specific training necessary to administer the study instruments to assess the outcome | 16-17 |
|  | 6a10 | Describe any processes used to promote outcome data quality during data collection (eg, duplicate measurements) and after data collection (eg, range checks of outcome data values), or state where these details can be found | NA |
|  | 6b | Any changes to trial outcomes after the trial commenced, with reasons | 11 |
| Sample size | 7a | How sample size was determined | 16 |
|  | 7a1 | Define and justify the target difference between treatment groups (eg, the minimal important difference) | NA |
|  | 7b | When applicable, explanation of any interim analyses and stopping guidelines | NA |
| Randomisation: |  |  |  |
| Sequence generation | 8a | Method used to generate the random allocation sequence | 16-17 |
|  | 8b | Type of randomisation; details of any restriction (such as blocking and block size) | 16-17 |
| Allocation concealment mechanism | 9 | Mechanism used to implement the random allocation sequence (such as sequentially numbered containers), describing any steps taken to conceal the sequence until interventions were assigned | 16-17 |
| Implementation | 10 | Who generated the random allocation sequence, who enrolled participants, and who assigned participants to interventions | 16-17 |
| Blinding | 11a | If done, who was blinded after assignment to interventions (for example, participants, care providers, those assessing outcomes) and how | 17 |
|  | 11b | If relevant, description of the similarity of interventions | NA |
| Statistical methods | 12a | Statistical methods used to compare groups for primary and secondary outcomes | 17-18 |
|  | 12a1 | Describe any methods used to account for multiplicity in the analysis or interpretation of the primary and secondary outcomes (eg, coprimary outcomes, same outcome assessed at multiple time points, or subgroup analyses of an outcome) | NA |
|  | 12a2 | State and justify any criteria for excluding any outcome data from the analysis and reporting, or report that no outcome data were excluded | NA |
|  | 12a3 | Describe the methods used to assess patterns of missingness (eg, missing not at random), and describe the methods used to handle missing outcome items or entire assessments | NA |
|  | 12a4 | Provide a definition of the outcome analysis population relating to nonadherence of the trial protocol (eg, as a  randomized analysis) | NA |
|  | 12b | Methods for additional analyses, such as subgroup analyses and adjusted analyses | NA |
| Results | | | |
| Participant flow (a diagram is strongly recommended) | 13a | For each group, the numbers of participants who were randomly assigned, received intended treatment, and were analysed for the primary outcome | 18-19 |
|  | 13b | For each group, losses and exclusions after randomisation, together with reasons | 18-19 |
| Recruitment | 14a | Dates defining the periods of recruitment and follow-up | 18 |
|  | 14b | Why the trial ended or was stopped | NA |
| Baseline data | 15 | A table showing baseline demographic and clinical characteristics for each group | Table 1; 19 |
| Numbers analysed | 16 | For each group, number of participants (denominator) included in each analysis and whether the analysis was by original assigned groups | Tables 1 to 4 |
| Outcomes and estimation | 17a | For each primary and secondary outcome, results for each group, and the estimated effect size and its precision (such as 95% confidence interval) | Tables 3 to 4 |
|  | 17a1 | Include the results for all prespecified outcome analyses or state where the results can be found if not in this report | 16; 20-22 |
|  | 17b | For binary outcomes, presentation of both absolute and relative effect sizes is recommended | NA |
| Ancillary analyses | 18 | Results of any other analyses performed, including subgroup analyses and adjusted analyses, distinguishing pre-specified from exploratory | NA |
|  | 18.1 | If there were any analyses that were not prespecified, explain why they were performed | NA |
| Harms | 19 | All important harms or unintended effects in each group (for specific guidance see CONSORT for harms) | 22 |
| Discussion | | | |
| Limitations | 20 | Trial limitations, addressing sources of potential bias, imprecision, and, if relevant, multiplicity of analyses | 28-30 |
| Generalisability | 21 | Generalisability (external validity, applicability) of the trial findings | 28-30 |
| Interpretation | 22 | Interpretation consistent with results, balancing benefits and harms, and considering other relevant evidence | 22-30 |
| Other information | | |  |
| Registration | 23 | Registration number and name of trial registry | 2 |
| Protocol | 24 | Where the full trial protocol can be accessed, if available | 2 |
| Funding | 25 | Sources of funding and other support (such as supply of drugs), role of funders | 2 |
